# Quantitative Estimation of Muscle Shear Elastic Modulus of the Upper Trapezius with Supersonic Shear Imaging during Arm Positioning

**DOI:** 10.1371/journal.pone.0067199

**Published:** 2013-06-25

**Authors:** Hio-Teng Leong, Gabriel Yin-fat Ng, Vivian Yee-fong Leung, Siu Ngor Fu

**Affiliations:** 1 Department of Rehabilitation Sciences, The Hong Kong Polytechnic University, Hung Hom, Kowloon, Hong Kong SAR, China; 2 Department of Imaging and Interventional Radiology, Prince of Wales Hospital, The Chinese University of Hong Kong, Shatin, New Territories, Hong Kong SAR, China; The University of Western Ontario, Canada

## Abstract

Pain and tenderness of the upper trapezius are the major complaints among people with chronic neck and shoulder disorders. Hyper-activation and increased muscle tension of the upper trapezius during arm elevation will cause imbalance of the scapular muscle force and contribute to neck and shoulder disorders. Assessing the elasticity of the upper trapezius in different arm positions is therefore important for identifying people at risk so as to give preventive programmes or for monitoring the effectiveness of the intervention programmes for these disorders. This study aimed to establish the reliability of supersonic shear imaging (SSI) in quantifying upper trapezius elasticity/shear elastic modulus and its ability to measure the modulation of muscle elasticity during arm elevation. Twenty-eight healthy adults (15 males, 13 females; mean age = 29.6 years) were recruited to participate in the study. In each participant, the shear elastic modulus of the upper trapezius while the arm was at rest and at 30° abduction was measured by two operators and twice by operator 1 with a time interval between the measurements. The results showed excellent within- and between-session intra-operator (ICC = 0.87–0.97) and inter-observer (ICC = 0.78–0.83) reliability for the upper trapezius elasticity with the arm at rest and at 30° abduction. An increase of 55.23% of shear elastic modulus from resting to 30° abduction was observed. Our findings demonstrate the possibilities for using SSI to quantify muscle elasticity and its potential role in delineating the modulation of upper trapezius elasticity, which is essential for future studies to compare the differences in shear elastic modulus between normal elasticity and that of individuals with neck and shoulder disorders.

## Introduction

Neck and shoulder disorders are common musculoskeletal problems, with more than 30% of the working population suffering from them [1]. Pain and tenderness of the upper trapezius with increase in muscle tension are the major complaints of people with chronic neck and shoulder problems. Hyper-activation and increased muscle tension of the upper trapezius during arm elevation will cause an imbalance of the scapular force couples and contribute to neck and shoulder pain [2], [3]. Assessing the elasticity of the upper trapezius in different arm positions is therefore important for identifying people at risk so as to give preventive programmes or for monitoring the effectiveness of intervention programmes for these disorders.

Supersonic shear imaging (SSI) is a newly developed technique that can be used to quantify soft tissue stiffness. This is done by using the ultrasound probe which emits focused beams to generate the acoustic radiation force to push the muscle and induce shear waves. By calculating the propagation speed of the shear wave, the shear elastic modulus can be estimated (in kPa) [4]–[8]. Significant increases in the muscle shear elastic modulus were reported during active contraction of the biceps [9] and gastrocnemius muscles [7], [10]. During the active contraction of the biceps muscle, a high correlation between the muscle shear elastic modulus and the percentage of maximum voluntary contractions (MVC) captured by electromyography (EMG) was observed in healthy adults [9]. Note that a greater percentage change of the muscle shear elastic modulus (of around 80%) was found when compared with the MVC (of around 20%). Such findings suggests that the muscle shear elastic modulus captured from SSI could be one of the tools for measuring muscle stiffness associated with muscle contraction, since it is able to show greater percentage changes when compared with EMG. This would be an advantage in measuring low muscle activation levels, such as that of the upper trapezius in the early movement of abduction [11], [12]. Nordez & Hug [9] also reported a good test-retest reliability of the shear elastic modulus during active muscle contraction when measurements were taken within the same session (ICCs ranged from 0.89 to 0.94). Between-session reliability during muscle contraction, however, is essential to monitor the progression of disease or the efficacy of intervention.

In view of the high incidence of neck and shoulder disorders that might be related to the changes in elasticity of the upper trapezius muscles, the aims of this study were: 1) to establish the intra- and inter-operator reliability of measuring the upper trapezius muscles’ elasticity using the SSI; and 2) to explore the feasibility of using SSI to delineate modulation in muscle stiffness of the upper trapezius associated with arm positioning in the early phase of abduction.

## Methods

### Ethics Statement

The study was approved in accordance with the guidelines of the Human Subjects Ethics Sub-committee by the ethical review of the Departmental Research Committee, The Hong Kong Polytechnic University, and all participants gave their written consent before the study. The subject of the photograph in the figures has given written informed consent, as outlined in the PLOS consent form, to publication of their photograph.

### Participants

Twenty-eight healthy individuals (15 males, 13 females, ranging in age from 18 to 55 years) were recruited from the community for this study. They were not taking steroids or muscle relaxants. Individuals with histories of shoulder fractures, instability or dislocation, shoulder surgery or clinical treatment for shoulder injuries were excluded.

### Supersonic Shear Imaging (SSI)

**Figure 1:**
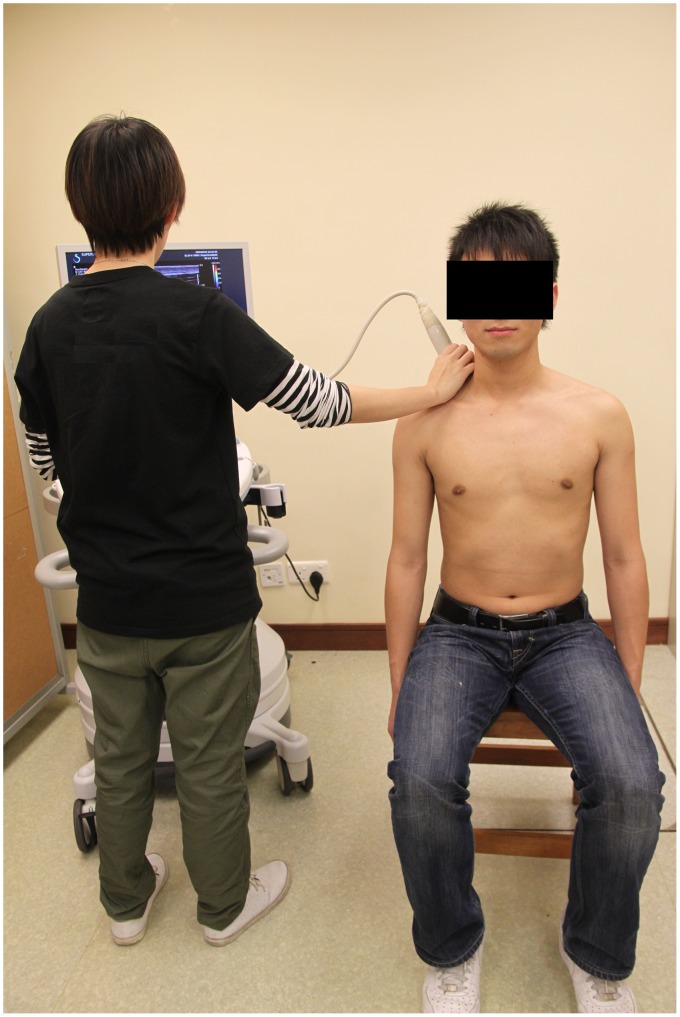
Subject position for the measurement of upper trapezius muscle elasticity with arm at rest.

**Figure 2:**
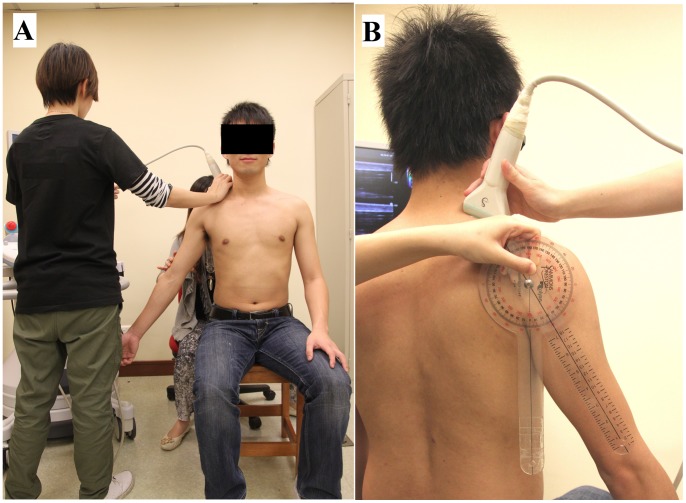
Subject position for the measurement of upper trapezius muscle elasticity with arm at 30° abduction. **(A) Front view; (B) Back view.**

Each subject was asked to sit upright on a stool with the head in a neutral position. The upper trapezius muscle elasticity of the dominant arm of each participant (defined as the writing arm) was measured in two shoulder positions: (1) in the resting position (Figure 1), and (2) after static holding at 30° of abduction for 10 seconds (Figure 2). In the resting position, the subject was asked to relax the arm with 0° of flexion, with the elbow in full extension and the forearm in a neutral position. During arm abduction, the subject was asked to abduct the shoulder to 30° with the elbow extended and the thumb pointing to the ceiling for 10 seconds. The angle of shoulder abduction was measured by a plastic goniometer (Sammons Preston, Royan, Canada). The abduction angle at 30° of arm elevation was chosen to assess the modulation of muscle stiffness associated with the active positioning of the arm in the early phase of the shoulder abduction.

**Figure 3:**
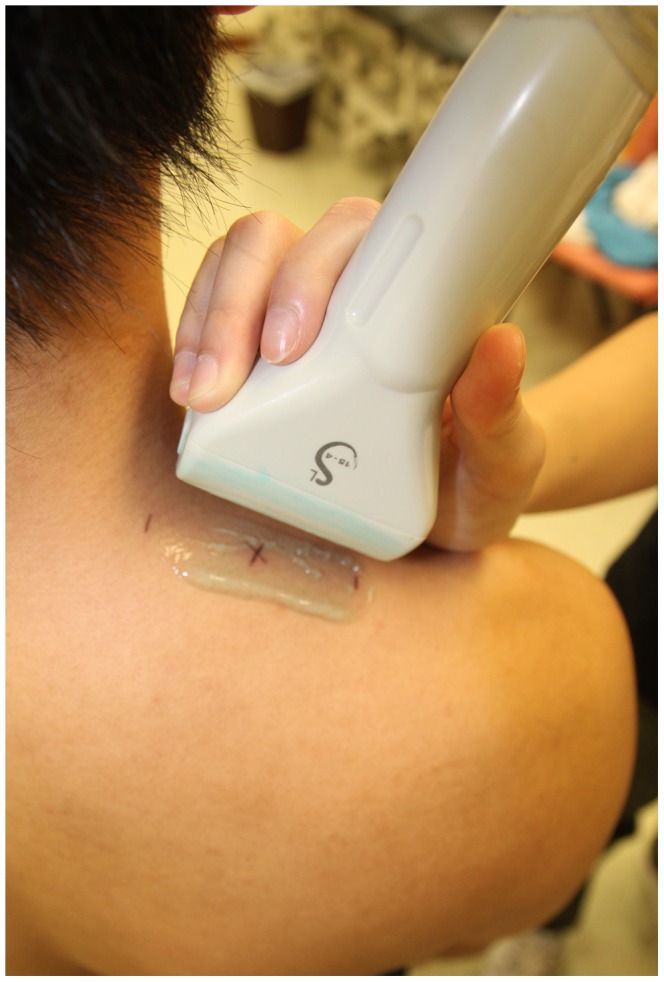
The probe was placed mid-way (marked with a ‘x’) between the angle of the acromion and the seventh cervical vertebra.

Each participant’s upper trapezius muscle was scanned with an Aixplorer **®** ultrasound scanning system (SuperSonic Imagine, Aix-en-Provence, France) with a bandwidth between 4 and 15 MHz and length of 55 mm linear-array transducer. The scanning site of the muscle was mid-way between the angle of acromion and the seventh cervical spine with reference to the recommended placement of the surface electrodes for electromyography (Figure 3) [13]. The location of the scanning site was measured with a measuring tape, and was marked with a pen on the skin by the operator in each session. The marking on the skin was then cleaned and erased after each session of scanning. The muscle was first scanned in the longitudinal view by grey-scale ultrasound to find the scanning site. Once the muscle was identified, the probe was placed parallel to the muscle fibers to avoid muscle anisotropic artifact [10], [14], and the shear wave elastography mode was then activated. The probe was placed perpendicularly on the skin with light pressure for 10 seconds to measure the shear elastic modulus, as recommended by the manufacturer [8]. Light pressure was defined as placing the transducer very lightly on top of a generous amount of coupling gel on the surface of the skin without deformation of the muscle thickness [8].

**Figure 4:**
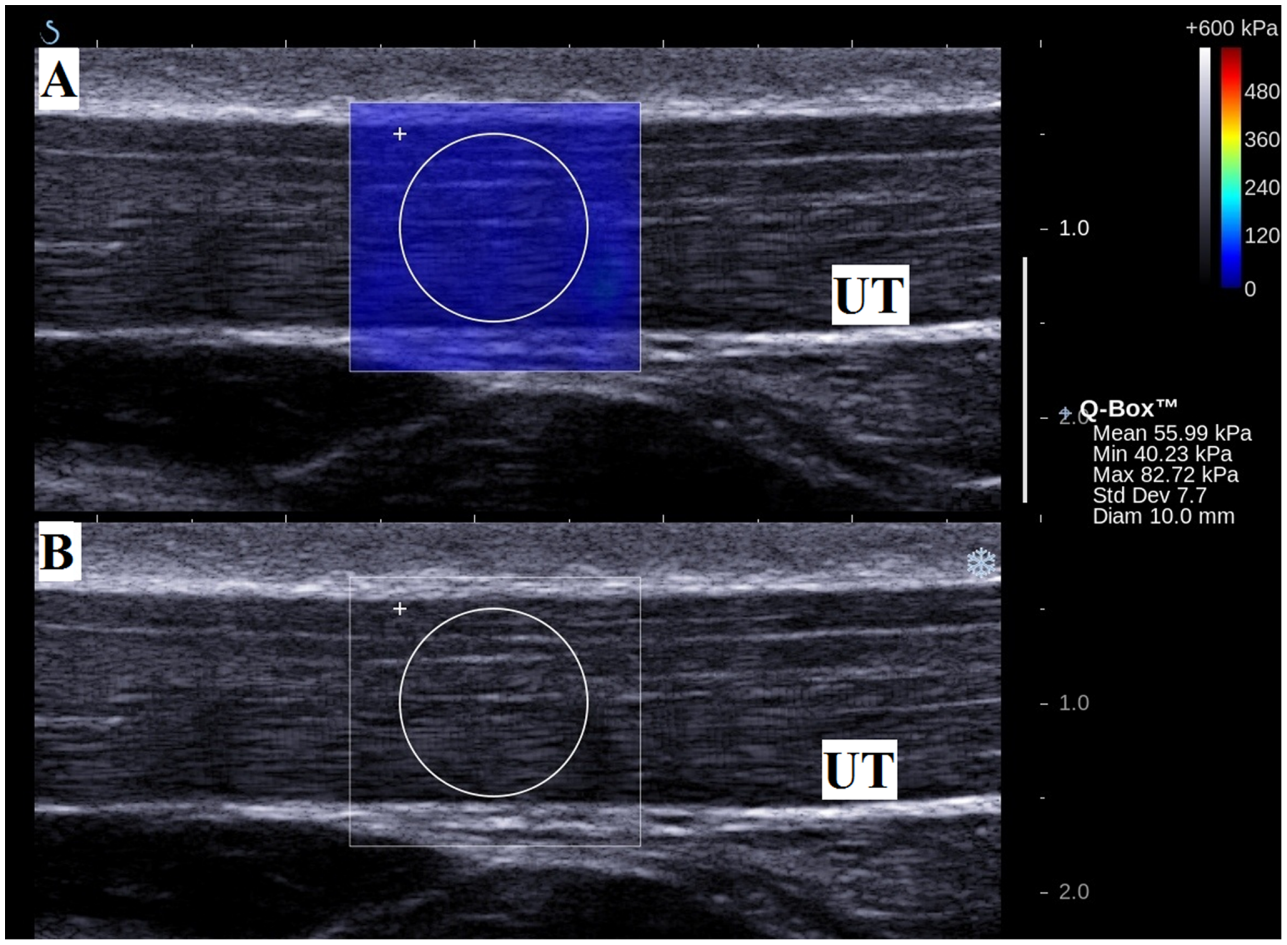
Measurement of elasticity of the upper trapezius muscle (UT) in longitudinal view. (A) Color-coded box presentations of the UT elasticity (stiffer areas were coded in red and softer areas in blue) superimposed on a longitudinal grey scale sonogram of the UT, with the circle representing the region of interest (ROI), and were adjusted according to the thickness of the muscle. The shear elastic modulus of the ROI is shown in the Q-box™ on the image. (B) Longitudinal grey scale sonograms of UT on the identical scan planes.

The machine was set to a standard mode, 50% opacity (or transparency) of the elasticity map over the B-mode imaging, and a 600 kPa scale color map was used. A circle that delineated the region of interest (ROI) for the measurement of the elastic modulus was placed at the center of the SSI acquisition box on the upper trapezius muscle (Figure 4). The size of the ROI was adjusted according to the thickness of the muscle [9]. Using a time-of-flight estimation, the shear wave velocity (Vs) along the probe was determined by a one-dimensional cross correlation of successive radio-frequency signals [5]. Then, considering a linear elastic behavior, the shear elastic modulus (µ) was calculated by the equation µ = ρVs^2^, where ρ was the density of muscle (1000 kg/m^3^) [5], [15]; it can be noted that shear elastic modulus increases with shear wave velocity [6]. However, the Aixplorer provides the Young modulus, thus, all the values obtained using the Aixplorer should be divided by 3 [15], [18]. The mean shear elastic modulus within the defined ROI was then generated. Three measurements were recorded in each arm position and a 1-minute rest was given between each measurement to avoid muscle fatigue. The shear elastic moduli in the three measurements in each arm position were averaged for statistical analysis.

### Reliability Assessments by Operators

Two operators (LHT and LVYF) participated in the reliability investigation. The first operator (LHT) is a sports physiotherapist with two years of experience in ultrasound scanning. The second operator (LVYF) is a sonographer with more than 20 years of experience in musculoskeletal ultrasound scanning. Before the study, both operators had studied how to use the SSI intensively for 3 months to become familiar with the measurement procedures.

There were two sessions in the intra-operator reliability test. In the first session, each subject was assessed by operator LHT according to the above protocol. Three measurements were taken to test for within-session intra-operator variation. In the second session, the same subject was rescanned by the same operator after a one-hour interval to test for any temporal variation. The average of the three measurements in session 1 and 2 was recorded to test the between-session intra-operator reliability.

To test for inter-operator reliability, each subject was assessed by both operators according to the above protocol, with 30 minutes between measurements. The operators were blinded to each other’s results during the test. The averaged values of the three measurements in each arm position assessed by each operator were recorded for statistical analysis.

### Statistical Analysis

Descriptive statistics were calculated for all demographic data. An intraclass correlation coefficient (ICC) with 95% confidence interval (CI) was calculated to determine the intra- and inter-operator reliability. The ICC (3,1) (two way mixed effect model, consistency) was calculated for the within- and between-session intra-operator reliability. ICC (2,2) (two way random effect model, absolute agreement) was determined for the inter-operator reliability. An ICC value of <0.40 is generally considered as poor reliability, between 0.40 and 0.75 as moderate to good, and a value of >0.75 indicates excellent reliability [16]. On the basis of the reliability coefficients, the standard error of measurement (SEM) was calculated (SEM = SD × √1-ICC) for each measurement. To compute the minimal detectable difference (MMD) as the 95% confidence interval limits of the SEM, the SEM was multiplied by 1.96 (for 95% interval) and by the square root of 2 for the difference values (1.96 × SEM × √2) [17]. A paired t-test was used to compare the averaged shear elastic moduli between the two arm positions, namely, at rest and in the active static holding at 30° abduction measured in Session 1. The statistical analyses were performed using SPSS Version 18 for Windows (SPSS Inc, Chicago, IL.) The level of significance (α) for all tests was set at 0.05.

## Results

### Reliability Analysis

The upper trapezius muscles of the dominant arm from 28 individuals (15 males, 13 females; mean age = 29.6±13.5 years) were assessed for within- and between-session intra-operator reliability. Of these, the upper trapezius muscles from 21 individuals were also assessed by both operators to establish inter-operator reliability. The intra- and inter-operator reliability and agreement of the mean shear elastic modulus of the upper trapezius muscles with the arm at rest and at 30° abduction are presented in Table 1. The ICC values show excellent repeatability for both within-session intra-operator measurements with the arm at rest (ICC = 0.97; 95% CI = 0.94–0.98) and at 30° abduction (ICC = 0.93; 95% CI = 0.88–0.97); and between-session intra-operator reliability with the arm at rest (ICC = 0.87; 95% CI = 0.72–0.94; MDD<5.81 kPa) and at 30° abduction (ICC = 0.95; 95% CI = 0.89–0.98; MDD<8.56 kPa). The ICC values also revealed excellent inter-operator repeatability while the arm was at rest (ICC = 0.78; 95% CI = 0.47–0.91; MDD<4.67 kPa) and at 30° abduction (ICC = 0.83; 95% CI = 0.59–0.93; MDD<17.26 kPa).

**Table 1:** Intra- and inter-operator reliability and agreement of the upper trapezius muscle elasticity with arm at rest and at 30° abduction in session 1 (a) and session 2 (b).

	Arm at rest			Arm at 30° abduction		
	Shear elastic modulus Mean ± SD (kPa)	SEM (kPa)	MDD (kPa)	Shear elastic modulus Mean ± SD (kPa)	SEM (kPa)	MDD (kPa)
**Intra-operator reliability (operator-LHT)**						
*a. Session 1* (28 shoulders)						
Within-session ICC (95% CI)	0.97 (0.94–0.98)			0.93 (0.88–0.97)		
*b. Session 2* (28 shoulders)						
Averaged value in Session 1	17.11±5.82	2.09	5.81	26.56±12.32	2.71	7.51
Averaged value in Session 2	16.28±4.04	1.46	4.03	27.71±14.04	3.09	8.56
Between-session ICC (95% CI)	0.87 (0.72–0.94)			0.95 (0.89–0.98)		
**Inter-operator reliability** (21 shoulders)						
Operator I (LHT, PT)	15.77±3.59	1.69	4.67	29.48±12.60	5.17	14.32
Operator II (LVYF, S)	15.00±3.35	1.57	4.36	32.00±15.19	6.23	17.26
ICC (95% CI)	0.78 (0.47–0.91)			0.83 (0.59–0.93)		

ICC: Intraclass correlation coefficient; CI: Confidence interval; SEM: Standard error of measurement; MDD: Minimal detectable difference; PT: Physiotherapist; S: Sonographer; kPa: kilo Pascal.

### Effects of Active Arm Abduction in Muscle Elasticity

**Figure 5:**
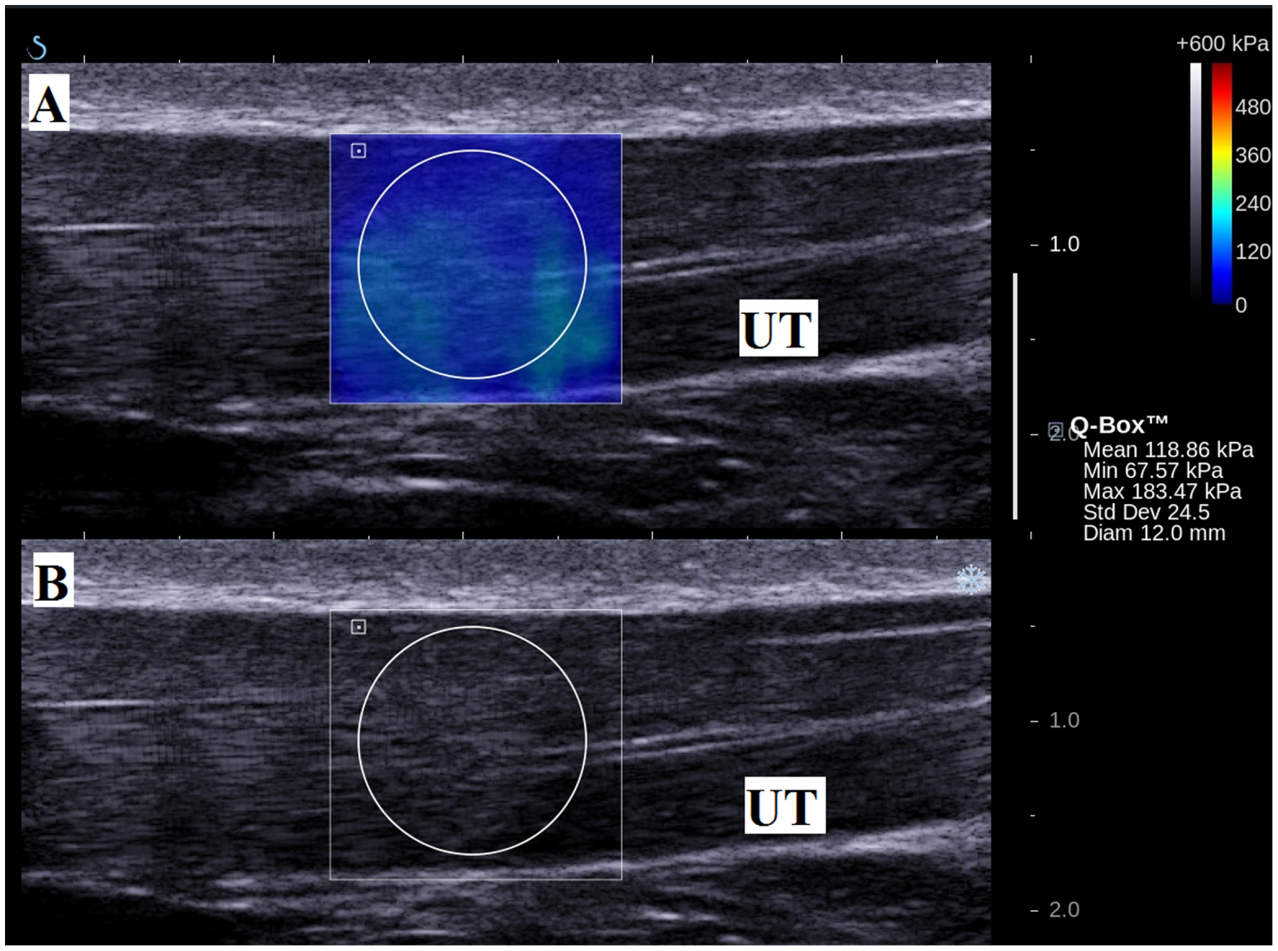
Measurement of elasticity of the upper trapezius muscle (UT) in longitudinal view with arm at 30° abduction. (A) Color-coded box presentations of the UT elasticity (stiffer areas were coded in red and softer areas in blue) superimposed on a longitudinal grey scale sonogram of the UT, with the circle representing the region of interest (ROI), and were adjusted according to the thickness of the muscle. The shear elastic modulus of the ROI is shown in the Q-box™ on the image. (B) Longitudinal grey scale sonograms of UT on the identical scan planes.

The shear elastic modulus of the upper trapezius muscle at rest was 17.11±5.82 kPa, and this increased to 26.56±12.32 kPa during active arm holding at 30° abduction (p = 0.00) (Figure 5). This revealed an increase of 55.23% in the shear elastic modulus from the resting position to the active arm abduction at 30°. This indicates that the SSI is able to capture modulation in the upper trapezius muscle elasticity during the early movement of arm abduction.

## Discussion

In the present study, we found excellent intra- and inter-operator repeatability in using SSI to measure the elasticity of the upper trapezius muscle, and that supersonic shear imaging is also able to capture the modulation of upper trapezius muscle stiffness associated with arm positioning.

### Reliability

Our results revealed excellent within-session intra-operator reliability for the measurements of elasticity of the upper trapezius with the arm at rest (ICC = 0.97) and at 30° abduction (ICC = 0.93) done by the same operator. This shows that SSI is a reliable technique for assessing muscle elasticity. Furthermore, we conducted the between-session reliability test to evaluate if there was any temporal variation. We found SSI to be reliable for measuring elasticity at 2 different time points. Our results have also revealed excellent inter-operator reliability with the arm at rest (ICC = 0.78) and at 30° abduction (ICC = 0.83). The reason for such high reliability in our study might be that we standardized the scanning site of the upper trapezius muscle with reference to body landmarks, the imaging parameters in using SSI, and the location and size of the (ROI) measurement, thus resulting in a more repeatable method for assessing muscle elasticity [8].

Our findings are comparable with those of previous studies showing good to excellent reliability in measuring muscle elasticity using the SSI in resting muscles and during active contraction [9], [18]. Lacourpaille et al. [18] examined the reliability of measuring the elastic modulus in nine resting muscles using the SSI and reported good intra-session reliability (Intraclass correlation coefficients (ICCs) = 0.87), inter-day reliability (ICC = 0.81) and inter-observer reliability (ICC = 0.71). In a study by Nordez and Hug [9], they used SSI to estimate muscle elasticity during active muscle contraction and found excellent repeatability the estimated elasticity of biceps muscles at both 3% and 7% of maximal EMG activity (ICC = 0.89 and ICC = 0.94, respectively). The findings from the present study have established good repeatability for using SSI to estimate the muscle elasticity of trapezius muscles when healthy subjects are requested to hold their arms in two different positions.

When compared with conventional elastography in which manual compression is applied with the ultrasound probe to estimate soft tissue stiffness, lower reliability was reported for the conventional technique, with ICCs ranging from 0.51 for plantar fascia to 0.76 for Achilles tendon [19], [20]. The disadvantages of this conventional elastography technique include operator dependence, as the variable compression applied by the user on the ultrasound probe can affect the outcome, and this technique can only generate qualitative rather than quantitative data [7], [21], [22].

Skeletal muscles are known to be highly anisotropic, and the measured wave speed is highly dependent on the probe orientation [10]. However, a recent study by Bouillard et al. [5] examined the effect of the muscle architecture of a bi-pennate muscle (first dorsal interosseous) and a fusiform muscle (abductor digiti minimi) and found no significant difference between muscles in the estimated contraction force using both SSI and EMG, which inferred that the pennation of muscle fibers does not influence the accuracy of estimation. In our study, the estimated shear elastic modulus for the upper trapezius was obtained through scanning in the longitudinal view and the probe was placed in parallel to the muscle fibers to avoid a muscle anisotropic artifact [10], [14].

Our study has also established the MDD values which represent the smallest difference in the shear elastic modulus of the upper trapezius muscle associated with a real change. This information is essential as it provides guidelines for interpreting changes in the upper trapezius muscle elasticity in different arm positions. On the basis of the results of the present study, we recommend using the averaged shear elastic modulus of three measurements in SSI assessed by the same operator to determine the muscle elasticity of the upper trapezius, and a mean change of 5.81 kPa in the arm resting position and 8.56 kPa during active arm abduction may assist in the interpretation of results to reflect real changes in future longitudinal studies.

### Modulation on Muscle Elasticity

Modulation of stiffness of the upper trapezius muscles associated with active holding of the shoulder could be delineated by the SSI in the present study. Indeed, the findings from the study indicated that the average shear elastic modulus of the upper trapezius muscle measured at rest was 17.11±5.82 kPa and was increased to 26.56±12.32 kPa at 30° of active shoulder abduction, and the change of the shear elastic modulus was greater than the MDD indicating real changes. Previous studies have reported low activation levels of the upper trapezius in the early movement of abduction in healthy individuals [11], [12], with the onset timing of this muscle measured by EMG being 0.067 seconds after movement onset, indicating that the upper trapezius is an upward scapular rotator and stabilizer during arm elevation [12]. Our results showed a significant increase of 55.23% in shear elastic modulus of the upper trapezius muscles during early arm abduction, indicating the contraction of this muscle during early humeral movement so as to provide stability of the scapula during arm elevation. In addition, studies have also shown increased upper trapezius activities in people with shoulder pathologies [23], [24], that may have been associated with decreased activities and delayed the onset time of serratus anterior and lower trapezius [25] and alterations in scapular kinematics [23], [24], suggesting that hyper-activation or increased muscle tension in the upper trapezius will cause imbalance of the scapular force couples and contribute to neck and shoulder pain [2], [3], [25]. Chen et al. [26] explored the upper trapezius muscle stiffness in people with myofascial pain by using Magnetic resonance elastography (MRE), and reported that the stiffness of the taut bands in people with myofascial pain (9.0±0.9 kPa) was greater than the surrounding musculature (6.2±0.8 kPa), with 50% greater than the healthy controls (4.1±0.6 kPa). Elastic modulus measured by SSI was found two times greater than by MRE [21], [26]. One possible explanation may be the positioning of the participants during measurements [27]. In the study of Chen et al. [26], the upper trapezius muscle elasticity was measured with the subject prone lying; however, in our study, we asked the subject to sit upright on a stool and the upper trapezius muscle elasticity was measured with the arm by the side. Viir et al. [27] demonstrated that changing from a sitting position to a supine position reduced the muscle tone and stiffness of the upper trapezius muscle by up to one-fifth indicating the important characteristics of the musculoskeletal support system.

Besides, our findings are comparable to those of previous studies demonstrating an increase in the elastic modulus during muscle contraction. Nordez & Hug [9] used the SWUE to estimate the muscle elasticity of the biceps muscles and showed significant linear regression between the muscle elasticity and EMG activity level. As well, Gennisson et al. [10] reported a spontaneous increase in the elastic modulus of brachialis muscles, from 5.9 kPa to 100.8 kPa when performing isometric contractions from resting to 4 kg loading. Shinohara et al. [7] examined the muscle elasticity of the lower limb muscles and also demonstrated an increase in muscle stiffness of the tibialis anterior, medial gastrocnemius, and soleus muscle when performing static dorsiflexion and plantarflexion with 30% of maximal voluntary contraction. The findings from our study demonstrated the potential use of SSI in assessing changes in the muscle elasticity of the trapezius muscles that is less expensive than MRE. It may provide a basis for assessing the muscle elasticity of the upper trapezius in healthy individuals, which is essential for future studies to compare the shear elastic modulus between normal and pathologic tissues.

### Limitations

There were some limitations of this study that need to be considered. (1) SSI yields an estimate of the effective shear elastic modulus of an assumed linear and purely elastic incompressible material and hence the effects of non-linearity, pre-strain, viscoelasticity and sources of anisotropy (e.g. fibrous content, pre-strain) are currently ignored. Assumptions were made about the biomechanical nature of the tissue and this is not meant to represent a “true” mechanical property but simply a measure in the domain of stiffness/elasticity which may be useful clinically. (2) The amount of physical activity of the participants was not recorded although muscle performance may affect the change in the elastic properties of the muscles. (3) Only the elasticity of the middle portion of the upper trapezius muscle was examined, as referenced to the recommended placement of surface electrodes for electromyography such that we could standardize the measuring method. Further studies are required to examine whether there are any differences in the shear elastic modulus between each portion of the upper trapezius muscle. (4) All participants were asymptomatic volunteers. Further studies could apply this technique to compare the results with those for symptomatic or pathological subjects.

### Conclusions

Supersonic shear imaging has excellent repeatability and reproducibility in determining the elasticity of the upper trapezius muscles. This imaging technique is sensitive for assessing the modulation of muscle stiffness associated with active positioning of the arm in the early phase of shoulder abduction. Its usefulness in classifying normal and pathological tissues and intervention efficacy, require further studies.
